# Candidate gene analysis supports a role for polymorphisms at *TCF7L2* as risk factors for type 2 diabetes in Sudan

**DOI:** 10.1186/s40200-016-0225-y

**Published:** 2016-03-01

**Authors:** Amir T. Ibrahim, Ayman Hussain, Mohamed A. M. Salih, Omima Abdeen Ibrahim, Sarra E Jamieson, Muntaser E. Ibrahim, Jenefer M. Blackwell, Hiba S. Mohamed

**Affiliations:** 1 Central Laboratory, Ministry of Science and Technology, Khartoum, Sudan; 2 Institute of Endemic Disease, University of Khartoum, P. O. Box 102 Khartoum, Sudan; 3 Tuti Health Centre, Khartoum, Sudan; 4 Telethon Kids Institute, University of Western Australia, Subiaco, Australia

**Keywords:** Type 2 diabetes mellitus, Genetic polymorphism, Genetic association study, Sudan

## Abstract

**Background:**

Genetic susceptibility to type 2 diabetes (T2D) is multifactorial. A growing number of genes have been identified as risk factors for T2D across multiple ethnicities in trans-ancestry meta-analysis of large-scale genome-wide association studies. Few studies have looked at these genes in Sub-Saharan African populations. This study was undertaken to look for associations between T2D and single nucleotide polymorphisms (SNPs) in a number of the top candidate genes in a selected Sudanese population.

**Methods:**

A total 240 T2D cases and 128 unrelated healthy control subjects were included in this study. Age, sex, weight and height were recorded, blood pressure and biochemical profiles of glucose and lipids were analysed. Single nucleotide polymorphism (SNP) genotyping was performed using the Sequenom MassARRAY® system. Fourteen SNPs were selected across 7 genes: *CAPN10* (rs2975760 and rs5030952), *PPARG* (rs17036314 and rs1801282), *IGF2BP2* (rs4402960 and rs1470579), *CDKAL1* (rs9465871), *HHEX* (rs1111875), *TCF7L2* (rs7903146, rs11196205 and rs12255372), and *KCNJ11* (rs5215, rs1800467 and rs5219). Allelic and haplotype association analyses were performed under additive models in PLINK. *P* ≤ 0.007 (=0.05/7 genes) was the *P*-value required to achieve correction for multiple testing.

**Results:**

A significant genetic association between the SNPs rs7903146 (odds ratio 1.69, 95 % confidence interval 1.21–2.38, *P* = 0.002) and rs12255372 (odds ratio 1.70, 95 % confidence interval 1.20–2.41, *P* = 0.003) at *TCF7L2* and T2D was found in Sudanese population. These associations were retained after adjusting for age, sex and BMI (e.g. rs7903146: odds ratio 1.70, *P*_adj:age/sex/BMI_ = 0.005). The strongest haplotype association (odds ratio 2.24; *P*_adj:age/sex/BMI_ = 0.0003) comprised the two point haplotype T_C across rs7903146 and rs11196205. Stepwise logistic regression demonstrated that SNP rs7903146 added significant main effects to rs11196205 or rs12255372, whereas the reverse was not true, indicating that the main effect for association with T2D in this population is most strongly tagged by SNP rs7903146. Adjusted analyses also provided support for protection from T2D associated with minor alleles at SNPs rs2975760 at *CAPN10* (odds ratio 0.44, 95 % confidence interval 0.20-0.97, *P*_adj:age/sex/BMI_ = 0.042) and rs1111876 at *HHEX* (odds ratio 0.60, 95 % confidence interval 0.39- 0.93, *P*_adj:age/sex/BMI_ = 0.022).

**Conclusions:**

Multiethnic associations between T2D and SNPs at *TCF7L2*, *CAPN10* and *HHEX* extend to Sub-Saharan Africa, specifically Sudan.

## Background

Type 2 diabetes (T2D) is emerging as an important international health problem. The International Diabetes Federation (IDF) report that 382 million people worldwide are living with diabetes, with a further 316 million with impaired glucose tolerance and therefore at high risk from the disease [1]. Evidence published in the IDF Diabetes Atlas [1] disproves the misconception that diabetes is a disease of the wealthy. Indeed, more than 80 % of people with diabetes live in low and middle-income countries, with emerging hotspots including countries in the Middle East and Sub-Saharan Africa [1]. In Sudan, Awad et al. [2] reported a prevalence of 3.4 % and considered diabetes as the commonest cause of hospital admission and morbidity due to a non- communicable disease. The highest prevalence is reported in northern Sudan [3] and the lowest in the western desert regions [4], with data reported in the IDF Diabetes Atlas [1] suggesting prevalence varying from 6-10 % and 60 % of deaths due to diabetes occurring in people under 60 years of age.

Since the introduction of genome-wide association studies (GWAS) a large number of genes have been shown to be associated with T2D at genome-wide significance (generally accepted to be *P* ≤ 5x10^−8^ [5]), including many that have been shown to have effects across multiple ethnicities [6–19] and in trans-ancestry meta-analysis of large-scale GWAS [20]. Only a few studies have looked at these genes in Arab [16, 18, 19, 21–23] or Sub-Saharan African populations [8, 17]. In this study we looked for association between 14 single nucleotide polymorphisms (SNPs) in 7 of the top GWAS genes (*TCF7L2*, *CDKAL1*, *HHEX*, *IGF2BP2*, *KCNJ11*, *PPARG* and *CAPN10*) and risk of T2D in Sudan.

## Methods

### Study area and population

The study was approved by the ethics committee of Institute of Endemic Diseases, University of Khartoum. Written informed consent was obtained from all cases and controls, all of whom were from the population of *Tuti* Island, Khartoum, Sudan descended from one large family from the *Mahas* tribe of northern Sudan. Ascertainment of cases was by identification of patients attending Tuti Health Centres for monthly follow up. Individuals were classified as having T2D when they met all of the following criteria: (1) diagnosed with T2D by a qualified physician; (2) on a prescribed drug treatment regimen for T2D; and (3) returned biochemical test results of a fasting plasma glucose level of at least 126 mg/dl (=7 mmol/l in SI units) based on the criteria laid by the World Health Organisation [24]. Healthy controls were selected from the general population of Tuti Island. All participants were consented for blood by venipuncture for DNA and biochemical tests (cf. below), and for recording of height and weight to determine body mass index (BMI). All participants answered a questionnaire approved by the ethics committee, and designed to collect socioeconomic data, as well as, age, sex, family history of diabetes and diagnosis.

### Lipid profile and glucose measurement

Blood samples were obtained in Fluoride Oxalate and EDTA tubes from all participants after an overnight (8-12-h) fast. Plasma was used for the determination of glucose, total cholesterol, high density lipoprotein (HDL), low density lipoprotein (LDL) and triglyceride (TG). All of these parameters were measured using spectrophotometry (Spectrum lab 22PC).

### DNA extraction and SNPs genotyping

The guanidine chloride-chloroform method was used for DNA extraction. SNP genotyping was carried out at Shanghai Benegene Biotechnology Co., Ltd (Shanghai, China) using the Sequenom MassARRAY® system. A total of 14 SNPs was selected for genotyping across 7 genes: *CAPN10* (rs2975760 and rs5030952), *PPARG* (rs17036314 and rs1801282), *IGF2BP2* (rs4402960 and rs1470579), *CDKAL1* (rs9465871), *HHEX* (rs1111875), *TCF7L2* (rs7903146, rs11196205 and rs12255372,), and *KCNJ11* (rs5215, rs1800467 and rs5219). SNP selection was based on top SNPs reported from a number of published candidate gene, GWAS or replication studies [8, 9, 12, 20, 25–29].

### Statistical analysis

Statistical comparisons of demographic (age, sex) and quantitative clinical variables (systolic blood pressure, diastolic blood pressure, BMI, fasting glucose, total cholesterol, HDL, LDL, and TG) between cases and controls were analysed in Prism5 using T- tests with Welch’s correction for unequal variances when applicable. All SNPs were tested for Hardy-Weinberg equilibrium. Linkage disequilibrium (r2 and D') between SNPs was determined using Haploview 4.1. Analyses to determine associations between SNPs and T2D were performed under additive, dominant and recessive models using logistic regression within PLINK [30] (http://pngu.mgh.harvard.edu/purcell/plink), with/without adjustment for age, sex and BMI as indicated. Only data for the additive model are provided here as in every case this model was adequate to explain the associations observed. Haplotype analyses were also performed within PLINK.

## Results

### Demographic and clinical profile of cases and controls

Demographic, clinical and biochemical parameters for 225 T2D cases (age range 20 to 89 years) and 129 healthy controls (age range 20 to 85 years) originally recruited into the study are presented in Table 1. As expected, significant differences in clinical traits associated with T2D (systolic blood pressure, fasting glucose, and total cholesterol) were observed between cases and controls. Age and BMI also differed between cases and controls. Since age, sex and BMI are known to be independent risk factors for T2D [1], we adjusted for these in our genetic analyses looking for associations between SNPs and T2D. A total of 186 cases and 106 controls with complete records for age, sex, and BMI were taken forward in these adjusted association analyses as presented below.

**Table 1:** Clinical and biochemical parameters of type 2 diabetes cases and controls

Trait	Controls	Cases	T test^a^
	Mean ± SD (n)	Mean ± SD (n)	*P*-value
Age (years)	48 ± 15 (118)	58 ± 13 (225)	<0.001*
Systolic BP (mmHg)	122 ± 18 (104)	132 ± 22 (202)	<0.001*
Diastolic BP (mmHg)	75 ± 13 (105)	77 ± 15 (204)	0.231
BMI (kg/m^2^)	27 ± 6 (107)	29 ± 7 (234)	0.017
Fasting glucose (mg/dl)	105 ± 32 (66)	155 ± 58 (124)	<0.001*
Cholesterol (mg/dl)	198 ± 72 (67)	171 ± 63 (125)	0.012
HDL (mg/dl)	76 ± 43 (59)	69 ± 43 (115)	0.287
LDL (mg/dl)	115 ± 58 (47)	104 ± 60 (95)	0.281
TG (mg/dl)	129 ± 75 (66)	105 ± 68 (125)	0.026

^a^T-Test with Welch’s correction for unequal variance where applicable; *indicates unequal variances. SD = Standard Deviation, n = number of the samples, BP = Blood pressure, BMI = Body Mass Index, HDL = High Density Lipoprotein, LDL = Low Density Lipoproptein, TG = Triglyceride

### Characteristic of SNPs in the study population

All SNPs were in Hardy-Weinberg equilibrium in the control sample (*p* > 0.05). Minor allele frequencies (MAF) for SNPs are provided in Table 2, and compared with MAF for Hapmap populations. Two SNPs, *PPARG* rs1801282 and *KCNJ11* rs1800467, were at MAF < 0.05 in our population and were removed from the association analyses. These low MAFs were consistent with low MAFs for these SNPs in all three HapMap populations. The remaining 12 SNPs were taken forward in the association analyses. The strict *P*-value needed to achieve statistical significance taking multiple testing into account is *P* = 0.004 (=0.05/12). However, this is conservative since multiple SNPs within genes showed some degree of linkage disequilibrium, as might be expected given that they were selected as top SNPs for association with T2D in other populations. Therefore, *P* ≤ 0.007 (=0.05/7 genes) was used as the *P*-value required to achieve correction for multiple testing.

**Table 2:** Details of SNPs used in this study

Gene	Chr	SNP	bp Location GRCh38	Minor Allele	MAF SDN^a^	MAF HapMap Populations		
					*N* = 129	CEU	HCB	YRI
*CAPN10*	2	rs2975760	240591746	C	0.07	0.16	0.11	0.01
		rs5030952	240603286	T	0.35	0.05	0.26	0.63
*PPARG*	3	rs17036314	12335246	C	0.26	0.20	0.22	0.18
		rs1801282	12351626	G	0.02	0.08	0.02	0
*IGF2BP2*	3	rs4402960	185793899	G	0.46	0.72	0.78	0.46
		rs1470579	185811292	A	0.28	0.72	0.74	0.13
*CDKAL1*	6	rs9465871	20717024	C	0.34	0.16	0.48	0.61
*HHEX*	10	rs1111875	92703125	A	0.25	0.42	0.69	0.13
*TCF7L2*	10	rs7903146	112998590	T	0.30	0.28	0.02	0.26
		rs11196205	113047288	G	0.48	0.58	0.96	0.14
		rs12255372	113049143	T	0.24	0.22	0	0.27
*KCNJ11*	11	rs5215	17387083	C	0.13	0.39	0.35	0.01
		rs1800467	17387284	G	0.03	0.05	0	0.01
		rs5219	17388025	T	0.08	0.39	0.39	0

^a^MAF – minor allele frequency for Sudan (SDN) healthy controls

Details include minor allele frequencies (MAF) in control subjects (*N* = 129) from the Sudanese (SDN) study compared with CEU, HCB and YRI HapMap populations. Chr = chromosome; bp Location = chromosomal base pair location of the SNP in Genome Reference Consortium Human Build 38 (GRCh38)

### Allelic association between candidate gene SNPs and T2D in Sudan

DNA was available for genotyping from 190 cases and 129 controls. Table 3 provides results of logistic regression analyses to test for allelic associations between SNPs at candidate genes and T2D. In the unadjusted analysis that included all study participants with DNA available (Table 3: Column entitled Unadjusted All) two SNPs at *TCF7L2,* rs7903146 (odds ratio 1.69, 95 % confidence intervals 1.21-2.38, *P* = 0.002) and rs12255372 (odds ratio 1.70, 95 % confidence intervals 1.20-2.41, *P* = 0.003), achieved *P*-values that withstand correction for multiple testing (*P* < 0.007). In the reduced data set that comprised individuals with complete data for age, sex, and BMI, the three SNPs at *TCF7L2* achieved nominal *P* < 0.05 (Table 3: Column entitled Unadjusted). Of the three SNPs, significance at *TCF7L2* rs7903146 achieved *P* < 0.007 following adjustment for age, or age and sex. This was retained after additionally adjusting for BMI, suggesting that this is a gene for T2D and not the result of confounding due to the strong correlation between BMI and T2D [1]. SNPs rs2975760 at *CAPN10* and rs1111875 at *HHEX* also achieved nominal *P* < 0.05 after adjustments for age with/without additional adjustments for sex, or sex and BMI.

**Table 3:** Association between SNPs and T2D in the Sudan study population

Chr	Gene	SNP	bp Location	A1	N	Unadjusted All				N	Unadjusted				Adj Age				Adj Age, Sex				Adj Age, Sex, BMI			
						OR	L95	U95	P		OR	L95	U95	P	OR	L95	U95	P	OR	L95	U95	P	OR	L95	U95	*P*
2	*CAPN10*	rs2975760	241531413	C	317	0.62	0.31	1.24	0.175	259	0.52	0.25	1.07	0.074	**0.42**	**0.19**	**0.92**	**0.030**	**0.44**	**0.20**	**0.96**	**0.040**	**0.44**	**0.20**	**0.97**	**0.042**
2	*CAPN10*	rs5030952	241542953	T	314	0.80	0.57	1.12	0.197	257	0.78	0.54	1.15	0.208	0.82	0.55	1.21	0.314	0.80	0.54	1.20	0.280	0.80	0.54	1.19	0.274
3	*PPARG*	rs17036314	12376995	C	300	0.96	0.67	1.38	0.824	242	1.12	0.74	1.71	0.596	1.18	0.75	1.84	0.474	1.14	0.73	1.78	0.558	1.16	0.74	1.81	0.517
3	*IGF2BP2*	rs4402960	185511937	G	317	0.86	0.63	1.18	0.349	260	0.93	0.66	1.31	0.684	0.93	0.65	1.33	0.693	0.95	0.66	1.35	0.767	0.95	0.66	1.36	0.777
3	*IGF2BP2*	rs1470579	185529330	A	316	1.01	0.72	1.42	0.959	258	1.02	0.70	1.49	0.924	0.99	0.66	1.46	0.941	1.00	0.68	1.49	0.986	1.02	0.68	1.51	0.930
6	*CDKAL1*	rs9465871	20717505	C	315	1.27	0.94	1.73	0.120	257	1.17	0.83	1.65	0.376	1.15	0.81	1.65	0.432	1.15	0.80	1.65	0.456	1.15	0.80	1.66	0.443
10	*HHEX*	rs1111875	94463132	A	319	0.71	0.50	1.01	0.060	261	0.68	0.45	1.02	0.060	**0.61**	**0.40**	**0.94**	**0.026**	**0.61**	**0.40**	**0.95**	**0.027**	**0.60**	**0.39**	**0.93**	**0.022**
**10**	***TCF7L2***	**rs7903146**	**114758599**	**T**	301	**1.69**	**1.21**	**2.38**	**0.002**	245	**1.62**	**1.11**	**2.37**	**0.013**	**1.75**	**1.18**	**2.61**	**0.006**	**1.81**	**1.21**	**2.70**	**0.004**	**1.79**	**1.20**	**2.67**	**0.005**
**10**	***TCF7L2***	**rs11196205**	**114807297**	**G**	306	0.78	0.58	1.06	0.117	248	**0.68**	**0.48**	**0.96**	**0.029**	**0.63**	**0.44**	**0.91**	**0.013**	**0.64**	**0.44**	**0.92**	**0.017**	**0.64**	**0.44**	**0.93**	**0.018**
**10**	***TCF7L2***	**rs12255372**	**114809152**	**T**	319	**1.70**	**1.20**	**2.41**	**0.003**	261	**1.53**	**1.04**	**2.25**	**0.031**	**1.57**	**1.06**	**2.33**	**0.026**	**1.58**	**1.07**	**2.35**	**0.023**	**1.60**	**1.08**	**2.38**	**0.021**
11	*KCNJ11*	rs5215	17408880	C	315	1.13	0.69	1.85	0.622	257	0.96	0.56	1.63	0.877	1.02	0.59	1.77	0.944	1.05	0.60	1.84	0.852	1.04	0.60	1.82	0.881
11	*KCNJ11*	rs5219	17409822	T	316	1.24	0.71	2.16	0.452	258	1.11	0.60	2.06	0.735	1.25	0.66	2.37	0.500	1.31	0.68	2.51	0.419	1.27	0.66	2.45	0.468

Data are presented for an unadjusted analysis for all participants in the study, and for unadjusted and adjusted (as indicated) analyses performed in the subset of individuals for whom complete data on age, sex and BMI was available. Chr = chromosome; bp Location indicates the chromosomal base pair location of the SNP in Genome Reference Consortium Human Build 38 (GRCh38); OR = odds ratio; L95 and U95 indicate lower and upper 95 % confidence intervals. Bold indicates data for SNPs with P<0.05

### Haplotype associations and stepwise logistic regression analysis for SNPs at *TCF7L2*

Since all three SNPs at *TCF7L2* were associated with T2D in our population, the question arises as to whether all SNPs were tagging a single functional variant, or whether multiple main effects occur. SNPs within *TCF7L2* were in strong linkage disequilibrium as determined by D' (rs7903146 and rs11196205: D' = 0.94; rs7903146 to rs12255372: D' = 0.68; rs11196205 to rs12255372: D' = 0.80), suggesting that all 3 SNPs may be tagging a single haplotype carrying the risk variant. This was not so strongly supported by linkage disequilibrium as determined by r^2^ (rs7903146 and rs11196205: r^2^ = 0.36; rs7903146 to rs12255372: r^2^ = 0.35; rs11196205 to rs12255372: r^2^ = 0.23) which takes allele frequencies into account. It was of interest too that the minor allele at rs11196205 was associated with protection, whereas for the other two *TCF7L2* SNPs the minor alleles were the risk alleles. We therefore carried out haplotype association analyses to see where the strongest associated haplotypes occur. As before (Table 3), the strongest single point association was at rs7903146 (odds ratio 1.70; *P*_adj:age/sex/BMI_ = 0.005). Notably, the associated (odds ratio >1) two point and three point risk haplotypes were on the background of the common C allele at rs11196205 (Table 4), with the strongest association (odds ratio 2.24; *P*_adj:age/sex/BMI_ = 0.0003) comprising the two point haplotype T_C across rs7903146 and rs11196205. In a stepwise logistic regression analysis (Table 5), SNP rs7903146 added significant main effects to rs11196205, whereas the reverse was not true. Similarly, whilst rs7903146 added significant main effects to rs12255372, the reverse was not true. Together these results are consistent with a single main effect for association with T2D in this population that is most strongly tagged by SNP rs7903146.

**Table 4:** The effects of multiple SNPs at *TCF7L2* on T2D risk in the Sudanese population

Allele/Haplotype	Frequency	rs7903146	rs11196205	rs12255372
T	0.398	OR = 1.79; *P* = 0.005		
C	0.567		OR = 1.56; *P* = 0.018	
T	0.33			OR = 1.60; *P* = 0.021
TC	0.377	**OR = 2.24;** ***P*** **= 0.0003**		
CT	0.294		OR = 1.86; *P* = 0.005	
TCT	0.236	OR = 1.86; *P* = 0.010		

Analysis undertaken under an additive logistic regression model (adjusted for age, sex, and BMI) to look for associations between haplotypes for the 3 SNPs at *TCF7L2* and risk of T2D in the Sudanese population. Bold indicates the most significant haplotype association

**Table 5:** The effects of multiple SNPs at *TCF7L2* on T2D risk in the Sudanese population

Null model	Alternative model	OR	P-value
rs7903146	rs7903146 + rs11196205	0.98	0.938
rs7903146	rs7903146 + rs12255372	1.22	0.437
**rs11196205**	**rs11196205 + rs7903146**	**2.04**	**0.010**
rs11196205	rs11196205 + rs12255372	1.53	0.078
rs12255372	rs12255372 + rs7903146	1.61	0.052
rs12255372	rs12255372 + rs11196205	0.77	0.219

Stepwise logistic regression analysis to determine whether SNPs at *TCF7L2* contribute separate main effects. A significant test comparing null and alternative models indicates that the marker added under the alternative model is contributing a separate main effect from the marker considered under the null hypothesis. Bold indicates nominal *P* < 0.05

## Discussion

In the present study, we investigated a possible association between T2D in Sudan and SNPs from 6 genes (*PPARG, IGF2BP2, CDKAL1, HHEX, TCF7L2,* and *KCNJ11*) that had achieved genome-wide significance in multiple GWAS undertaken across different ethnicities [31], in addition to *CAPN10* which had been shown to be associated with T2D in a large study of Caucasians [26] and in Tunisia [27]. Of these, we found significant evidence for association between T2D and SNPs at *TCF7L2* in our Sudanese population, and suggestive evidence for associations at SNPs in *CAPN10* and *HHEX*. Given the limitations of our sample size, we cannot discount the possibility that further associations with SNPs in more of these genes would be found in this population if larger sample sizes were employed. However, relatively few studies have determined the role of common global T2D associated genetic variants in Sub-Saharan Africa, and to our knowledge this is the first study to provide evidence for associations between T2D and these important diabetes genes in a Sudanese population. Thus our study contributes to the growing need to replicate associations observed in GWAS carried out predominantly in Caucasian populations.

*CAPN10* is of some interest as the first T2D gene to have been identified in a genome wide linkage scan [32], followed by positional cloning [33]. Subsequent large-scale meta-analysis of 13,628 subjects found only a modest effect size (odds ratio 1.15; 95 % confidence intervals 1.07-1.23; *P* = 0.0002) for allele T at SNP rs2975760 as a risk factor for T2D [26], and no GWAS have reported *P* < 1x10^−5^ for association between T2D and *CAPN10* as currently recorded in the NCBI Catalog of published GWAS [31]. Some evidence for associations with larger effect sizes (odds ratios 1.35 to 1.61) were recently reported for SNPs at *CAPN10* and risk of T2D in a Tunisian Arab population [27]. Here we show that the T allele at SNP rs2975760 was also associated with a larger effect size (odds ratio 2.38; 95 % confidence interval 1.09-5.26; *P* = 0.03) in the Sudanese study population following adjustment for age. Further work is required to determine whether this is indicative of population-specific roles for polymorphisms at *CAPN10* as risk factors for T2D in African populations.

The association at *HHEX* is also of interest given prior strong candidacy of this gene as a risk factor for T2D in multiple other populations [8, 9, 20], in this case well supported by results of multiple GWAS including trans-ancestry studies recorded in the NCBI Catalog of published GWAS [31]. HHEX belongs to a large family of transcription factors that are distinguished by a 60 amino acid conserved DNA-binding homeodomain. It is expressed in the anterior visceral endoderm during early development and in some adult tissues of endodermal origin, including liver and thyroid. In humans HHEX is associated with decreased insulin secretion in response to oral glucose stimulation, while knockout mouse studies demonstrate that the gene is a regulator of glucose-stimulated insulin secretion [34]. *HHEX* is located in a region of chromosome 10 that harbors several genes involved in beta-cell function or development, including the *TCF7L2* gene. However, the two genes are located some 10 Mb apart, and their effects were independent in our study.

Associations observed between polymorphisms at *TCF7L2* and T2D were the most strongly supported in our Sudanese study population. This is in keeping with GWAS and replication studies providing evidence for a role for this gene as a risk factor for T2D across multiple ethnicities [10–12, 14, 20, 31, 35], including a growing number of studies in African Americans [15], Sub-Saharan African [8, 17, 36] and Arab [16, 19, 21, 23, 25, 37–39] populations. Indeed, *TCF7L2* was once described as the biggest story in diabetes since HLA [40]. Exceptions include studies in Pima Indians [41] and an Australian Aboriginal population [42]. This may relate to sample size, and/or to the observation across several studies that *TCF7L2* variants are associated with reduced BMI in diabetes cases (but not in controls) [40]. Hence, in a population where T2D is predominantly associated with high BMI, the association with *TCF7L2* might not be so readily observed. In Emiratis, for example, association between *TCF7L2* and T2D was only observed in a non-obese case group and not in obese diabetics [38]. In our Sudanese study, our sample size was too small to stratify by BMI, but our association between *TCF7L2* variants and T2D was robust to adjustment for BMI.

*TCF7L2* encodes a high-mobility group box-containing transcription factor. Collective evidence (reviewed [43]) supports the hypothesis that WNT/β-catenin signaling via TCF7L2 regulates Glucagon-like peptide 1 effects in pancreatic β-cells by transcriptionally regulating its receptor. In humans, the risk T allele at rs7903146 (the SNP most strongly associated with T2D in our Sudanese population) strongly predicted future T2D in two independent cohorts, and was associated with impaired insulin secretion, incretin effects, and an enhanced rate of hepatic glucose production [44]. TCF7L2 expression in human islets was increased 5-fold in T2D, particularly in carriers of the TT genotype. Overexpression of TCF7L2 in human islets reduced glucose-stimulated insulin secretion. These authors conclude that the increased risk of T2D conferred by variants in *TCF7L2* involves the enteroinsular axis, enhanced expression of the gene in islets, and impaired insulin secretion [44].

## Conclusions

In this study we found significant evidence for association between T2D and SNPs at *TCF7L2* in our Sudanese population, and suggestive evidence for associations at SNPs in *CAPN10* and *HHEX*. The latter require confirmation in studies of larger sample sizes in Sudan, and we cannot exclude the possibility of associations with other genes examined here. Given its strong global associations with T2D, the demonstration of association between *TCF7L2* and T2D in this Sudanese population is important. The cumulative evidence for associations between *TCF7L2* polymorphisms across multiple ethnicities, together with growing knowledge of WNT/TCF7L2 signaling in β-cells, make it an attractive target for development of novel therapies for diabetes [43]. Our research demonstrates that T2D patients in Sudan would likely benefit from this translation of genetic research to the clinic.

### Ethics approval and consent to participate

The study was approved by the ethics committee of Institute of Endemic Diseases, University of Khartoum. Written informed consent was obtained from all cases and controls.

### Availability of data

Summary data are available from the corresponding author as appropriate for meta-analyses.

## Abbreviations

T2D: Type 2 diabetes
SNP: Single nucleotide polymorphisms
BMI: Body mass index
IDF: International Diabetes Foundation
GWAS: Genome-wide association study
DNA: Deoxyribonucleic acid
HLD: High density lipoprotein
LDL: Low density lipoprotein
TG: Triglyceride
MAF: Minor allele frequency
